# Midwifery

**Published:** 1838-01

**Authors:** 


### MIDWIFERY.

### *On Extra-uterine Pregnancy.* By J. E. Dezeimeris, m.d.

Respecting the seat of this abnormal conception, ten varieties are enumerated: —1st. Ovarian pregnancy. 2d. Sub-peritoneo-pelvic. 3d. Tubo-ovarian. 4th. Tubar. 5th. Tubo-abdominal. 6th. Tubo-utero-interstitial. 7th. Utero-interstitial. 8th. Utero-tubar. 9th. Utero-tubo-abdominal. 10th. Abdominal; primitive, secondary. M. Dezeimeris raises, *in limine*, the question of the possibility or impossibility of ovarian pregnancy. MM. Prevost and Dumas have proved the important office of the spermatic animalculæ in the act of fecundation, but not that their actual introduction into the ovule is necessary to vivification; nor yet that these animalculæ, imprisoned within the cavity of the womb, wait there for the descent of an ovule. On the contrary, tubal pregnancies are incontestible and uncontested; and, as it is not to be supposed that the fœtus developed in the tube had been fecundated in the womb, and had thence reascended to its narrow abode, so must its vivification in the fallopian tube be admitted by all. This first step being gained, the succeeding will be made with comparative facility. Even those who deny the possibility of impregnation taking place while the ovule is yet enveloped by the investment of the ovaries, must yet admit some vivifying influence that induces the ovule to burst its boundary walls and descend through the tube. Of the second variety are cited two cases where a fœtus was found between the layers of the broad ligaments, one of them dissected by Professor Lobstein. Among all the cases upon record of utero-interstitial pregnancy, the most valuable for the authenticity of its details is that communicated by Dance to M. Breschet. It is evident in this case that the pregnancy was really and purely interstitial, that is, without the participation of the fallopian tube *in* the wall of the cyst containing the fœtus; the tube, in traversing the uterus, was connected with the wall of the cyst, and presented in one point an opening, which was indeed taken for a rupture made in detaching the placenta, but whose existence more probably preceded the passage of the fœtus into the substance of the uterus. This circumstance of the case conducts us by insensible gradations from the tubo-utero-interstitial pregnancy to the simple interstitial, whose locality is more distant from the tube, and very much diminishes the mystery with which some have invested this state of parts.

Primitive abdominal pregnancy is interesting only as shewing upon what parts the fœtus is ingrafted, and how it supplies itself with the necessary quantity of blood. Secondary abdominal pregnancy involves the discussion, whether, as M. Guillemot states, some cases of normal gestation may not thus terminate, in consequence of rupture of the walls of the uterus: in either case, if the immediate danger be surmounted, the mother may sustain her unborn offspring for an unlimited period in this new condition.

*Pathological Anatomy of the Extra-uterine Fœtation.* In these cases the embryo generally retains its proper membranes, viz. the chorion and amnios, and also the placenta, if it has survived the first days of its existence: the placenta is larger than natural, thin, furnished with very small vessels; circumstances induced by the difficulty of obtaining an adequate supply of blood from the neighbouring organs. In the primitive abdominal pregnancy, there is rarely any enveloping cyst that can be considered analogous to the caducus; owing, no doubt, to the trifling inflammation produced in the first instance by the presence, in the cavity of the peritoneum, of so small a body. In the secondary form this cyst is always found, being indeed as necessary a consequence in this case as its absence was to be anticipated in the last: for, whether the fœtus be disengaged into the abdominal cavity, by rupture of its envelope, as a tubal, ovarian, or other gestation, or by rupture of the uterine walls, the presence of so large a foreign body could not but excite inflammation, the glueing together of the neighbouring organs, and thus at last a perfect cyst. As to the fœtus itself, 1, a remarkable development of the osseous system has been observed in some instances, as well as the presence of several teeth; 2, a putrid state of the fœtus, the bones of which made their exit from the body by different routes; 3, a dessicated or mummified condition; and, 4, its transformation into a chalky mass, into amazone, or into bone. Examples of monsters in these situations are rare.

*Of the Mother.* The normal change in the size and vascularity of the uterus, its gradual diminution and return to the condition of vacuity, as well as the formation of the membrana decidua, are attested by cases. The secretion of milk and the menstruation obey the usual laws of natural gestation: most uneasiness to the mother is occasioned by tubal and utero-interstitial fœtation: this condition offers no material obstacle to natural gestation and delivery. At the expiration of the ordinary period, childbed pains supervene, and last some days, and are often renewed at pretty regular intervals as long as the pregnancy continues.

*Journal des Connaissances Med. Chir. Janv.* 1837.

---

### *Unconscious Delivery.* By M. LEONHARD.

THE following case is related chiefly as of importance in a medico-legal point of view, in its bearings on the following questions: 1. Whether a female may entirely mistake the nearness of delivery? 2. Whether she places herself unintentionally in a place unfitted for delivery? 3. Whether, whilst the pregnant woman is fully possessed of consciousness, a delivery, of which she is unconscious, may take place?

Mrs. K—, aged thirty-seven, the mother of six living children, and having thrice miscarried, became pregnant for the tenth time, and calculated the term of her pregnancy during the first fortnight of May. Early in March she was exposed to the contagion of small-pox, and acquired the disease in a very violent form. A laxative clyster had been ordered on account of constipation of three days' continuance; but it was not administered, as, during the afternoon, the patient complained of a disposition to evacuate the bowels. She was raised upon the night-stool, and became suddenly and at once freed from this constipation. She remained, however, for a quarter of an hour in the same situation, because she continued to feel some desire to evacuate fæces, of which but a small quantity was passed. As she began to feel very faint, she was returning to bed, when, greatly to her astonishment, she found herself connected by a band with the night-stool, which she could not separate. This was discovered by an attendant to be connected with a child, which began to cry on being withdrawn from the bloody water. The female, who had given birth previously to six living children, could scarcely trust her eyes. *She had been entirely unconscious of what had preceded delivery, and had throughout perceived no indications of it.* The child was separated from the navel string, and the woman returned to bed. She died suddenly in about half an hour afterwards. The uterus had contracted; the secundines were loose in the vagina; that portion of umbilical cord which remained attached to it about an ell long. The child was a female, and appeared fully formed. From these observations, made in the case of a sensible and honest woman, and in the presence of several witnesses, the questions given in the first part of this paper may be answered thus: that a pregnant female, even though she has frequently borne children previously, may entirely mistake the approach of delivery,—may be unintentionally surprised in a situation and in circumstances the most inappropriate for it,—and may, even though quite conscious, be unconscious of the birth of her offspring. The importance of this case in some questions respecting illegitimate pregnancy, is evident to all.

[We cannot believe in the full consciousness of this patient during the passage of the fœtus: the case is, however, both curious and important.]

*Medicinische Zeitung.* No. 24. 1837.

---

### *Speculum Uteri made of Glass.*

DR. HACKER, of Leipzig, uses a glass speculum in examining the vagina and womb. It consists of a simple oval cylinder, the glass of which is about two lines in thickness, its length being from four to six inches. The diameter of the end which is introduced into the vagina is half or three-quarters of an inch by a quarter or half an inch. This extremity is furnished with a blunt, rounded, and tumid border, whilst the cylinder at the other end is funnel shaped. Its form corresponds essentially with that of other specula, except that its circumference is less. The advantages of these specula of glass are their cheapness, and the facility with which they can be cleansed. When, too, they are introduced into the vagina, its mucous membrane, immediately before the tumid extremity of the speculum, is pressed into the cavity of the speculum, where it can well be examined. If the material be transparent glass, by means of a bright artificial light, the parts of the vagina which are covered by the speculum may be examined. Specula of dark glass are better fitted for examining the mouth of the womb.

*Jahrbücher der in-und-ausländischen Medicin.* Heft 3. 1837.

---

*Instances of Menstruation occurring in Old Age.*

1. MADAME K. had ceased to menstruate in her forty-second year, and, with the exception of some attacks of gout, had continued well to her eightieth year. At this time (1832), attended with pains like colic, the menses returned, continuing, as formerly, for about four days, and regularly recurring until August, 1835. From that time they disappeared. The individual died in the beginning of 1836. According to her own statement, the secretion which escaped was, in every respect, of the same sensible characters as that of her earlier life.

*Med. Zeitung.* No. 48. 1836.

2. A female, who has never had children, menstruated regularly until her fiftieth year: from this period, onwards, there appeared every three months, at the time of the previous menstruation, an excretion of from ten to fifteen drops of blood. The woman is now seventy-four years of age, is very healthy, and continues to menstruate in the same manner.

*Jahrbücher der in-und-ausländischen Medicin.* Heft 1. 1837.

---

*Rupture of the Cæcum during Labour.* By Dr. STAMPF, of Stargard.

THE wife of an officer, at the commencement of her first pregnancy, was much distressed with vomiting, but it went off at the end of a week, and she suffered no remarkable inconvenience during the remainder of the gestation, excepting in the eighth month, when venesection was necessary to relieve a determination of blood to the head. The labour went through its stages in the usual way, and the child was born; but both the mother and husband of the patient declared they noticed a peculiar noise in the abdomen at the time of the last labour-pain, which was a very strong one. The first twenty-four hours after the birth passed away without any particular occurrence beyond sharp after-pains and a slight uneasiness in the groin. From this period, fever, with a hard, full pulse, frequent nausea and vomiting, considerable thirst, severe pains in the abdomen upon the slightest touch, unconquerable costiveness, and, in short, all the symptoms of inflammation, made their appearance, more referrible to the intestinal canal than the uterus and peritoneum. Frequent bleedings, &c. &c. were resorted to in vain; the patient died seventy-two hours after delivery.

Upon dissection, it was ascertained that the cæcum was ruptured in a diagonal direction, to the extent of nearly two inches, and that half a wash-hand basin of fæces had escaped from the opening into the abdominal cavity. The parts in the vicinity of the rupture were of normal character and free from inflammation, excepting on some parts of the intestine and peritoneum in the right inguinal region; but nowhere was there any evidence of mortification. The cavity of the abdomen and the intestines were much distended with flatus.

*Berliner Medicinische Zeitung.* 20 *April,* 1836.

### *Vagitus Uterinus before the Rupture of the Membranes.* By Dr. C. F. Dressel.

As Dr. D. was sitting one evening with his wife, aged thirty-one, who was pregnant for the third time, he heard two evident cries from the child in the mother's womb. At the same moment his wife looked at him alarmed, placed her hand upon her right side, and declared that the cry, which she also had heard, had proceeded from that spot. The doctor immediately applied his hand to the part, and felt the child move. In the moment of the cry the child had moved violently, and had caused a perceptible pressure on the right side of the scrobiculus cordis. The mother had been healthy throughout the whole pregnancy; there were no signs of delivery, which first commenced forty-eight hours afterwards, and ended by the birth of a fine girl. This case Dr. D. relates as a new instance of the possibility of the crying of a child in the womb before the evacuation of the waters.

*Jahrbücher der in-und-ausländischen Medicin.* Heft 1. 1837.

---

### *On Injections into the Vessels of the Umbilical Cord, in Cases of retained Placenta.* By Dr. De Berghes.

[This paper may be regarded as a sequel to one on the same subject in a former Number of this Journal, Vol. III., p. 244, to our remarks on which we beg to refer.]

The author has had several opportunities of witnessing the advantageous effects of injections of cold water into the veins of the navel-string, where the placenta is slow in being delivered. He relates four cases which fell under his own immediate notice. In all, the females had passed their fortieth year; they were strong, although lean; and in all of them the mammæ were but little developed. The first case is that of a woman who had borne eight children, and where, three hours after delivery, the placenta was still retained. The after-pains were few and feeble, the abdomen tumid, the discharge of blood trifling, and the os uteri sufficiently patent. By cutting off a small portion of the navel-string, it was easy to find an umbilical vessel, and to introduce into it the point of a common clyster syringe, with which a pint of cold water was injected. A sensation of chilliness and an increase of the after-pains were produced. A ligature was then applied to the navel-string, and the hand introduced into the womb, where the placenta was found everywhere adherent. The volume of the placenta, in spite of the injected water, was not large; and the fact of its being injected, and with cold water, very much facilitated its delivery.—The second case was somewhat similar, except that the placenta was but partially adherent.

In the third case, where the injection was made about two hours after the birth of the child, and in the fourth half an hour after the child was born, the placenta followed about eight minutes after the injections had been made, and without any assistance. In all the females the subsequent process was undisturbed, and the author is not aware of any injurious effects resulting from the treatment.

*Wochenschrift für die gesammte Heilkunde.* No. 86. 1837.

